# Associations of eHealth Literacy with Obtaining Knowledge about Colorectal Cancer among Internet Users Accessing a Reputable Cancer Website: Internet-Based Survey Study

**DOI:** 10.3390/ijerph17093302

**Published:** 2020-05-09

**Authors:** Seigo Mitsutake, Ai Shibata, Kaori Ishii, Rina Miyawaki, Koichiro Oka

**Affiliations:** 1 Human Care Research Team, Tokyo Metropolitan Institute of Gerontology, Tokyo 173-0015, Japan; 2 Waseda Institute for Sport Sciences, Waseda University, Saitama 359-1192, Japan; 3 Faculty of Health and Sport Sciences, University of Tsukuba, Ibaraki 305-8574, Japan; shibata.ai.ga@u.tsukuba.ac.jp; 4 Faculty of Sport Sciences, Waseda University, Saitama 359-1192, Japan; ishiikaori@waseda.jp (K.I.); koka@waseda.jp (K.O.); 5 School of Arts and Letters, Meiji University, Tokyo 168-8555, Japan; rina_miyawaki@meiji.ac.jp

**Keywords:** Colorectal cancer, cancer knowledge, eHealth literacy, health education, Internet

## Abstract

Examining the associations of eHealth literacy (eHL) with obtaining health knowledge from websites would help to clarify the causal pathway between eHL and health knowledge. This study aimed to compare the results obtained from Internet users with high or low eHL in accessing a reputable cancer website to obtain colorectal cancer (CRC) knowledge. A total of 105 participants with high eHL and 103 participants with low eHL accessed a reputable CRC website managed by the National Cancer Center and responded to Internet-based surveys before and after accessing a website in 2012. Twelve responses to knowledge statements regarding CRC were selected based on item response theory, and the differences in correct responses of pre- and post-surveys by each eHL group were compared. Two statements showed a significant increase in correct responses in the high eHL group only: “Red meat intake is a risk factor” *(p* = 0.002), and “Obesity is a risk factor” *(p* = 0.029), whereas only one response did so in the low eHL group: “Bloody stools are a symptom” *(p* = 0.004). Low eHL Internet users appeared less capable of obtaining knowledge of CRC by accessing information from a reputable cancer website than high eHL Internet users.

## 1. Introduction

The Internet has become a powerful source for influencing health behavior, knowledge of health, and medical care. According to an estimate from a communications usage trends survey in 2016, 83.0% of the general population had used the Internet in Japan [1]. It has also been reported that approximately 70% of Japanese Internet users have sought health information on the Internet [2]. Health information provided on the Internet has therefore become a more notable influence in health promotion among the general public through the widespread use of personal computers and smart phones.

However, a serious issue has emerged in that many websites providing health information are unreliable and may be linked more to the promotion of commercial goods or private health services [3,4]. To utilize health information on the Internet effectively, people seeking health information need to have eHealth literacy (eHL), that is, the ability to seek, find, understand, and appraise health information from electronic sources and apply the knowledge gained to addressing or solving a health problem [4].

Identifying the causal pathway whereby health knowledge is obtained from health information available on the Internet may help in developing useful websites for Internet users to enhance their health knowledge. According to research conducted through the Integrative Model of eHealth Use (IMeHU), eHL plays an essential role in improving health knowledge by facilitating website use [5]. Cross-sectional studies have shown a positive association between eHL, healthy behaviors, and correct knowledge of health [6,7,8,9,10,11,12,13,14]. However, a recent review of studies on the association between eHL and health outcomes indicated that a few studies on eHL and health outcomes did not find a causal pathway [15]. Moreover, there has been little research on whether eHL is associated with obtaining health knowledge from websites that provide health information.

Examining the association of eHL with obtaining health knowledge from health-related websites would help to clarify the causal pathway between eHL and health knowledge. The Integrated Model of health literacy of the Consortium Health Literacy Project European shows that the process of accessing, understanding, appraising, and applying health information has generated health knowledge among individuals [16]. Though one study has shown a positive association between eHL and knowledge of colorectal cancer (CRC) [7], to the best of our knowledge, no previous studies have examined whether eHL is associated with obtaining CRC knowledge via accessing information about CRC on the Internet. Based on the IMeHU and Integrated Model of health literacy, we hypothesized that low eHL Internet users may be less capable of obtaining knowledge of CRC by accessing information from a reputable website. It is crucial for adults (especially those aged 40–60 years) to gain knowledge about CRC to promote preventive behavior for CRC (i.e., annual screening practice), as the prevalence of CRC increases among adults aged over 40 years [17]; moreover, over 90% of Japanese adults aged 40–60 use the Internet [1]. Therefore, this study aimed to compare the results obtained on CRC knowledge by accessing a reputable cancer website among Internet users with high and low eHL.

## 2. Materials and Methods

### 2.1. Participants

In this study, we conducted pre- and post-surveys of selected participants who had used a website providing reliable cancer information. The participants were recruited from registered individuals of a Japanese Internet research service company called MyVoice Communication, Inc. For the surveys, Internet users were selected because eHL is only necessary for those who access online health information. The Internet research company was able to gather a representative sample among Internet users at a low cost. The research company had approximately 1,180,000 individuals registered in 2012, and it obtained detailed sociodemographic data on registration.

First, for the pre-survey phase (12–16 January 2012), the research company randomly chose potential respondents (*n* = 3307) from among its registered individuals aged 40–59 years (*n* = 461,160) and sent an e–mail invitation for participation in the survey ( Supplementary Figure S1). We sought to minimize potential selection bias due to proportional differences in sex and age. We therefore allocated potential respondents equally to four groups of 250 participants, each categorized by sex and age: male participants aged 40–49, female participants aged 40–49, male participants aged 50–59, and female participants aged 50–59. A total of 1069 individuals responded to the pre-survey invitation to participate in the survey (response rate: 32.3%, *n* = 1069/3307). The pre-survey respondents were divided into two groups: (1) 640 individuals with high eHL and (2) 429 individuals with low eHL groups using the median value of the Japanese eHealth Literacy Scale (J-eHEALS) (median = 23.5) [2,7].

For the post-survey phase (30 January–1 February 2012), to collect data from 200 participants (divided into two groups of 100 participants with high eHL and 100 participants with low eHL), the Internet research service company randomly chose 130 pre-survey respondents from each of the two eHL groups from among the 1069 respondents, according to the company’s response rate data. The company sent 260 pre-survey respondents an e-mail address including the URL of the “Cancer Information Service” managed by the National Cancer Center of Japan [17], which provides reliable cancer information. This e-mail instructed the participants to access the post-survey after searching for information on CRC using the Cancer Information Service. The research company constructed the survey program so that that the participants were not able to respond to the post-survey before accessing the Cancer Information Service. The relevant CRC information provided by the Cancer Information Service covered twelve sections in 2012, namely: the definition of CRC, the epidemiology of CRC, the symptoms of CRC, the process of CRC diagnosis, the tests identifying CRC, the stages of CRC, the cures for CRC, the complications of CRC, the precautions needed with CRC, the follow-up of CRC, the metastases of CRC, and the reoccurrence of CRC. The participants were able to access the information on CRC provided by the Cancer Information Services through using search engines or entering the words “colorectal cancer” and following the links. A total of 214 participants (response rate: 82.3%, *n* = 214/260) comprising 107 participants from each eHL group accessed the Cancer Information Service and gave responses to the post-survey. Finally, participants with a history of cancer were excluded because they would have more knowledge and interest about cancer than those without a history of cancer, following which 103 and 105 participants in the low eHL and high eHL groups, respectively, were analyzed.

The research company used a blinded selection process to select the potential respondents to ensure anonymity. The surveys were placed in a protected area of the website, and the potential respondents received a specific URL in their invitation e-mail. Potential respondents were able to log on to the protected area of the site using a unique ID and password. After the desired number of participants for the pre-survey and post-survey phases of this study had voluntarily signed an online informed consent form and completed the sociodemographic data information form, further participants were no longer accepted.

### 2.2. Measurements

The J-eHEALS was used to assess the eHL levels of the participants [2,18]. The data were collected at the pre-survey phase. The J-eHEALS involved the use of a 5-point Likert scale (ranging from 1: “strongly disagree” to 5: “strongly agree”; score range: 8–40) to measure perceived eHL among participants. To determine the validity of the J-eHEALS, confirmatory factor analysis was conducted using the data obtained for the present survey [2]. The analysis of an eight-item model suggested a good fit for the proposed model (Goodness of Fit Index = 0.988, Comparative Fit Index = 0.993, and Root Mean Square Error of Approximation = 0.056), and the internal reliability of the test was confirmed using Cronbach’s alpha coefficient = 0.93 [2].

Knowledge of CRC was assessed through correctly identifying 20 true/false statements regarding the definition, risk factors, screening prevention and symptoms of CRC. This self-administered test was adapted from previous studies of knowledge and attitudes concerning CRC [7,19,20,21]. The data were collected at both the pre- and post-survey phases.

The research company provided sociodemographic data on sex, age, marital status, educational attainment, and household income level. These data had been collected from individuals when they first registered with the company. The data were categorized as follows: sex (men, women); age groups (40–49 years, 50–59 years); marital status (not married, married); education level (up to high school, two years of college or career college, college graduate or above); and household income level (< 5 million yen, ≥ 5 million yen; USD 1= JPY 80, in 2012) [22]. We assessed the frequency of information searches on the Internet in terms of searches conducted daily. The data were collected at the pre-survey phase of the study.

### 2.3. Statistical Analyses

First, we employed a chi-square test to evaluate the differences in sociodemographic factors (sex, age, marital status, educational attainment, and household income level) and frequency of Internet searching between those with low and high eHL. Second, item selection of the knowledge of CRC was conducted based on the 2-parameter logistic Item Response Theory (IRT). The discrimination and difficulty parameters estimated by IRT can potentially provide information for improving the precision and reliability of an item in the tool. We selected those items with a discrimination parameter of above zero [23]. Third, the Wilcoxon signed-rank test was conducted to compare the differences in the proportion of correct responses to 12 items based on IRT selected during the pre- and post-survey phases. We used this test as the data of the proportion of correct responses to the 12 items were not a normal distribution. Fourth, to determine the association between eHL and knowledge of CRC after accessing information from the Cancer Information Service, a McNemar test was utilized to compare the differences in the proportions of correct responses for each CRC knowledge statement between the pre- and post-survey phase in the low and high eHL groups. In all analyses, a *p*-value < 0.05 was considered statistically significant. We used SPSS 25.0 (IBM Corp., Armonk, NY, USA) and STATA 16.0 (STARA Corp LP, TX, USA) to compute the statistics.

### 2.4. Ethics Approval and Consent to Participate

This study was approved by the Human Research Ethics Committee of Waseda University (Application No. 2011-245) and conformed to the Declaration of Helsinki. Entering the survey was defined as informed consent.

## 3. Results

### 3.1. Participant Characteristics 

Table 1 shows the characteristics of those with high or low eHL. In this study, the mean age (standard deviation, SD) of the total number of participants was 49.1 (5.5) years, and approximately 50% of the participants were men. Approximately 42% had graduated from college or graduate school and 64% had a household income < 5 million yen. Approximately 74% of the participants were married and 70% used the Internet for searching every day. There was no significant difference in characteristics between the high and low eHL groups. The mean J-eHEALS score was 22.7 (SD = 6.5) among all participants, 27.7 (SD = 3.8) among the high eHL group, and 17.6 (SD = 4.3) among the low eHL group.

### 3.2. Item Selection

Table 2 shows each CRC statement selected based on IRT. Twelve items were selected according to a discrimination parameter of above 0. The discrimination parameters of all chosen items ranged from 0.13 to 2.62. The difficulty of all items selected ranged from −14.41 to 0.66.

### 3.3. Response Analysis

**Figure 1:**
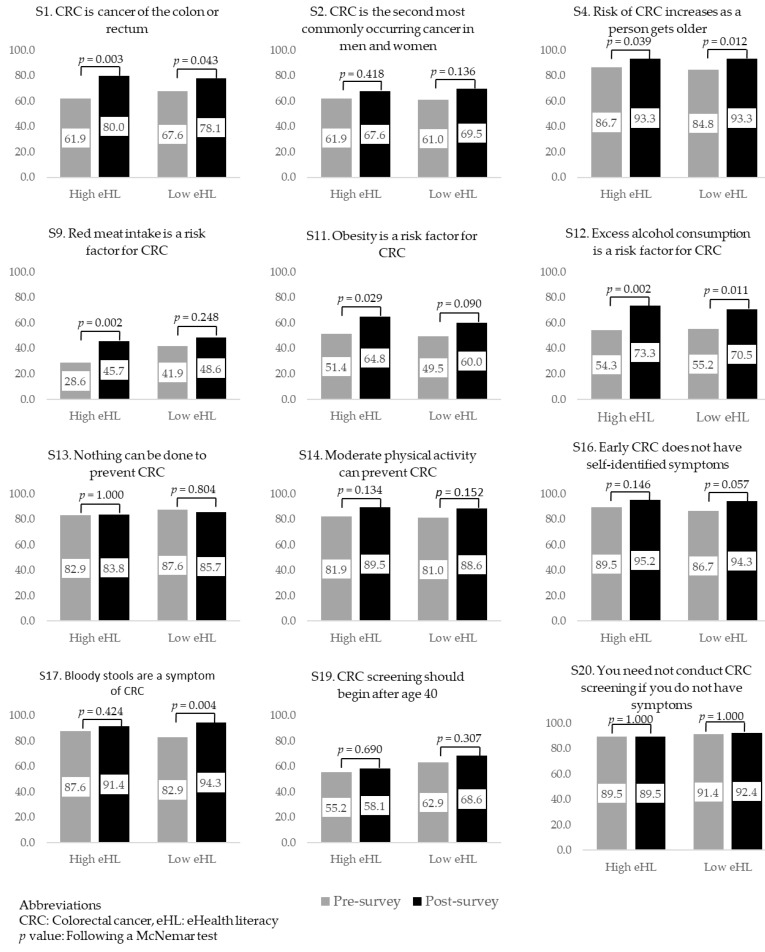
The differences in the proportion of correct responses for colorectal cancer (CRC) knowledge statements between the pre- and post-survey phases in the high and low eHealth literacy (eHL) groups.

The proportion of correct responses to the twelve items was higher in the post-survey (median: 83.3, 25 percentile–75 percentile: 66.7–91.7) than that in the pre-survey (median: 75.0, 25 percentile–75 percentile: 58.3–83.3, *p* < 0.001) among all 208 participants. Figure 1 presents the differences in the proportions of correct responses for CRC knowledge statements between the pre- and post-surveys in the high and low eHL groups. Three statements showed a significant increase in correct responses in both eHL groups in the post-survey: “S1. CRC is cancer of the colon or rectum” (high eHL: *p* = 0.003, low eHL: *p* = 0.043), “S4. The risk of CRC is greater as a person gets older” (high eHL: *p* = 0.039, low eHL: *p* = 0.012), and “S12. Excess alcohol consumption is a risk factor for CRC” (high eHL: *p* = 0.002, low eHL: *p* = 0.011). Two statements showed a statistically significant increase in correct responses in the high eHL group only: “S9. Red meat intake is a risk factor for CRC” (*p* = 0.002), and “S11. Obesity is a risk factor for CRC” (*p* = 0.029), whereas only one response did so in the low eHL group: “S17. Bloody stools are a symptom of CRC” (*p* = 0.004). Moreover, there were seven statements for which there was no significant change in those giving correct responses between the pre- and post-survey phases in the high eHL group, and eight statements in which no significant change in the numbers giving correct responses was noted between the pre- and post-survey phases in the low eHL group.

## 4. Discussion

To the best of our knowledge, this is the first study to compare the results of accessing, understanding, and appraising information on CRC obtained from a reputable cancer website on subsequent knowledge concerning CRC between Internet users with high and low eHL. While the correct response rate for five statements increased after using the Cancer Information Service among individuals with high eHL, the correct response rate for four statements increased among those with low eHL.

Our results showing that there was a higher correct response rate after using the Cancer Information Service among those with high eHL also accorded with research undertaken by the IMeHU and with prior studies [5,6,7,8,9,10,11,12,13,14]. According to the IMeHU, eHL plays an important role in enhancing health knowledge through facilitating website use [5]. This study showed that eHL may be a factor associated with improving CRC knowledge resulting from the use of a reputable cancer website. Internet users with low eHL were shown to be less likely than those with high eHL to improve their knowledge of cancer through accessing, understanding, and appraising the information from the reputable cancer website. Therefore, it is important to develop and improve reputable cancer websites that facilitate Internet users with low eHL to access, understand, and appraise relevant information. Additionally, it has been about eight years since the data for the present study were collected. Although a prior study developed an effective e-learning program on eHL in a randomized controlled trial [24], this program may not have been sufficiently diffused for general Internet users in Japan to gain eHL. Therefore, it is necessary to consider strategies for the diffusion of eHealth literacy for general Internet users.

Because the participants in this study had been provided with the URL to access the Cancer Information Service, they had less need of the ability to seek and appraise CRC information from various websites. eHL is considered to involving three dimensions (functional, interactive, and critical) based on a prior study using a different conceptualization of eHL than this study [9]. Functional eHL is more relevant for understanding CRC information available on reputable cancer websites than interactive and critical eHL [9]. Therefore, because the results of this study were more likely to have been obtained from participants with functional eHL, it appears that a focus on the functional eHL of Internet users may be useful for developing more effective cancer information websites. For example, because functional eHL involves basic reading skills and does not assume a sophisticated knowledge of health conditions and health systems [9], a reputable cancer website needs to provide information using plain language without technical jargon.

Moreover, Internet users with low functional eHL in this study may have been unable to find, access, and appraise reputable cancer websites on the Internet because the critical eHL needed for this requires more complex skills than functional eHL. One study has shown that those with more developed critical eHL engaged better in health-promoting activities than those with functional eHL [9]. Because the use of critical eHL is likely to help with obtaining cancer knowledge more effectively through using websites than functional eHL, it would be helpful to examine the associations between using critical eHL as well as functional eHL and obtaining cancer knowledge on reputable cancer websites. Therefore, future research is needed, using a modified survey program to find, access, and appraise the use of critical eHL in searching reputable websites, as well as to examine the association of critical eHL with obtaining cancer knowledge on the Internet.

The present study had several limitations. First, the participants were recruited from a single Japanese Internet research service company. While these participants were suitable for an Internet-based survey, as Internet users need to have adequate eHL, the results may have been biased because of the potentially unrepresentative nature of this sample in relation to general Japanese Internet users aged between 40 and 59 years old. Because this study comprised individuals registered with a research service company who were frequent Internet users, the participants of the present study may have had disproportionately higher eHL skill levels [2,25,26]. Therefore, this study may have underestimated the differences found concerning the extent of CRC knowledge obtained through the cancer website between those with high eHL and those with low eHL, compared to what might have been found in a study of general Internet users. Additionally, because the data used in this study came from existing Internet users registered with an Internet-survey company, the participants in this study were those with higher education levels and household incomes and were more frequent Internet users than the general population [2]. In this study, a comparison of households earning above and below 5 million yen per annum showed no difference in terms of high and low eHL, unlike in a prior study using a research service company [2]. Second, the sample size was too small to conduct a multivariable analysis adjusting for covariables. Therefore, we should consider the fact that the differences in the proportion of correct responses for CRC knowledge between the pre- and post-surveys in this study was not adjusted by confounders. Moreover, the small sample size may not be sufficiently powered to detect the differences in the proportion of correct responses for knowledge between the pre- and post-survey. Therefore, though a statement “S16. Early CRC does not have self-identified symptoms” showed a borderline significance increase in correct response (*p* = 0.057) in low eHL group in this Internet-based study, this statement could show a statistically significant increase in correct responses in the future study with a large general population-based study. Third, the present study did not objectively assess the dimensions of eHL. Therefore, inaccuracies in estimating participants’ eHL levels were unavoidable. Previous studies have objectively evaluated the eHL of participants using performance tests [27,28], and future research examining the effects of eHL on enhancing health knowledge also needs to use such methods. Finally, the responses of this study were derived from CRC information provided by the Cancer Information Service in 2012. An Internet user with low eHL can currently obtain more knowledge of CRC using the Cancer Information Service than was possible in 2012, as the website has since been improved to include more graphics and easier-to-understand language.

## 5. Conclusions

In this web-based study, Internet users with low eHL may be less able to obtain knowledge of CRC accessing a reputable cancer website than Internet users with high eHL. It is important to develop reputable cancer websites better adapted for the varying eHL abilities of Internet users to ensure that appropriate and reliable cancer knowledge can be obtained. Moreover, to develop robust evidence on the causal pathway between eHL and health knowledge, future research is needed to determine whether improving eHL helps to find a reputable website and understand and appraise health information on the Internet using data from the general population.

## Figures and Tables

**Table 1:** Participant characteristics (%) in the high eHealth literacy (eHL) and low eHL groups.

Characteristics		Total *n* = 208	High eHL Group *n* = 105	Low eHL Group *n* = 103	*p*-Value ^b^
Sex	Men	50.0	49.5	50.5	0.890
	Women	50.0	50.5	49.5	
Age groups	40–49	50.0	49.5	50.5	0.890
	50–59	50.0	50.5	49.5	
Education level	≤ High school graduate	27.4	26.7	28.2	0.474
	Two years of college or career college	30.8	27.6	34.0	
	≥ College graduate	41.8	45.7	37.9	
Household income (yen) ^a^	< 5 million	36.1	33.3	38.8	0.409
	≥ 5 million	63.9	66.7	61.2	
Marital status	Not married	26.0	25.7	26.2	0.935
	Married	74.0	74.3	73.8	
Frequency of Internet searching	Every day	70.2	74.3	66.0	0.193
	Not every day	29.8	25.7	34.0	

^a^, $1 = 80 yen, in 2012; ^b^, Chi-square test.

**Table 2:** Colorectal cancer (CRC) knowledge questions and answers.

CRC Knowledge Questions	Answers	Correct Answer (%)	IRT	
			Discrimination	Difficulty
Selected items				
S1. CRC is cancer of the colon or rectum	C	65.4	0.15	−4.17
S2. CRC is the second most commonly occurring cancer in men and women	I	62.0	0.43	−1.18
S4. Risk of CRC increases as a person gets older	C	86.5	1.18	−1.94
S9. Red meat intake is a risk factor for CRC	C	35.6	1.11	0.66
S11. Obesity is a risk factor for CRC	C	51.0	2.16	−0.03
S12. Excess alcohol consumption is a risk factor for CRC	C	55.3	2.63	−0.15
S13. Nothing can be done to prevent CRC	I	86.1	0.09	−19.74
S14. Moderate physical activity can prevent CRC	C	82.2	1.41	−1.43
S16. Early CRC does not have self-identified symptoms	C	88.9	1.24	−2.10
S17. Bloody stools are a symptom of CRC	C	86.1	1.32	−1.77
S19. CRC screening should begin after age 40	C	59.6	0.79	−0.56
S20. You need not conduct CRC screening if you do not have symptoms	I	91.3	0.24	−9.75
Unselected items				
S3. CRC is the leading cause of cancer-related death	I	71.6	−0.51	1.92
S5. Risk of CRC is the same for men and women	I	53.8	−0.03	5.00
S6. CRC is usually hereditary	I	88.5	−0.92	2.54
S7. Bowel infection is a risk factor for CRC	I	55.3	−0.84	0.29
S8. Cigarette smoking is a risk factor for CRC	I	51.0	−1.82	0.03
S10. Hypertension is a risk factor for CRC	I	74.0	−2.58	0.75
S15. Dietary fiber intake can prevent CRC	I	9.6	−2.25	−1.63
S18. Polakisuria is a symptom of CRC	I	84.1	−0.76	2.44

Abbreviations: CRC, colorectal cancer; IRT, Item Response Theory; C, Correct; I, Incorrect.

## Supplementary Materials

The following are available online at https://www.mdpi.com/1660-4601/17/9/3302/s1, Figure S1: Flowchart of the participants.
