# Supplementary figures and images for: Associations of eHealth Literacy with Obtaining Knowledge about Colorectal Cancer among Internet Users Accessing a Reputable Cancer Website: Internet-Based Survey Study
Source: Int J Environ Res Public Health. 2020 May 9;17(9):3302. doi: 10.3390/ijerph17093302 (PMC7246812; doi:10.3390/ijerph17093302)

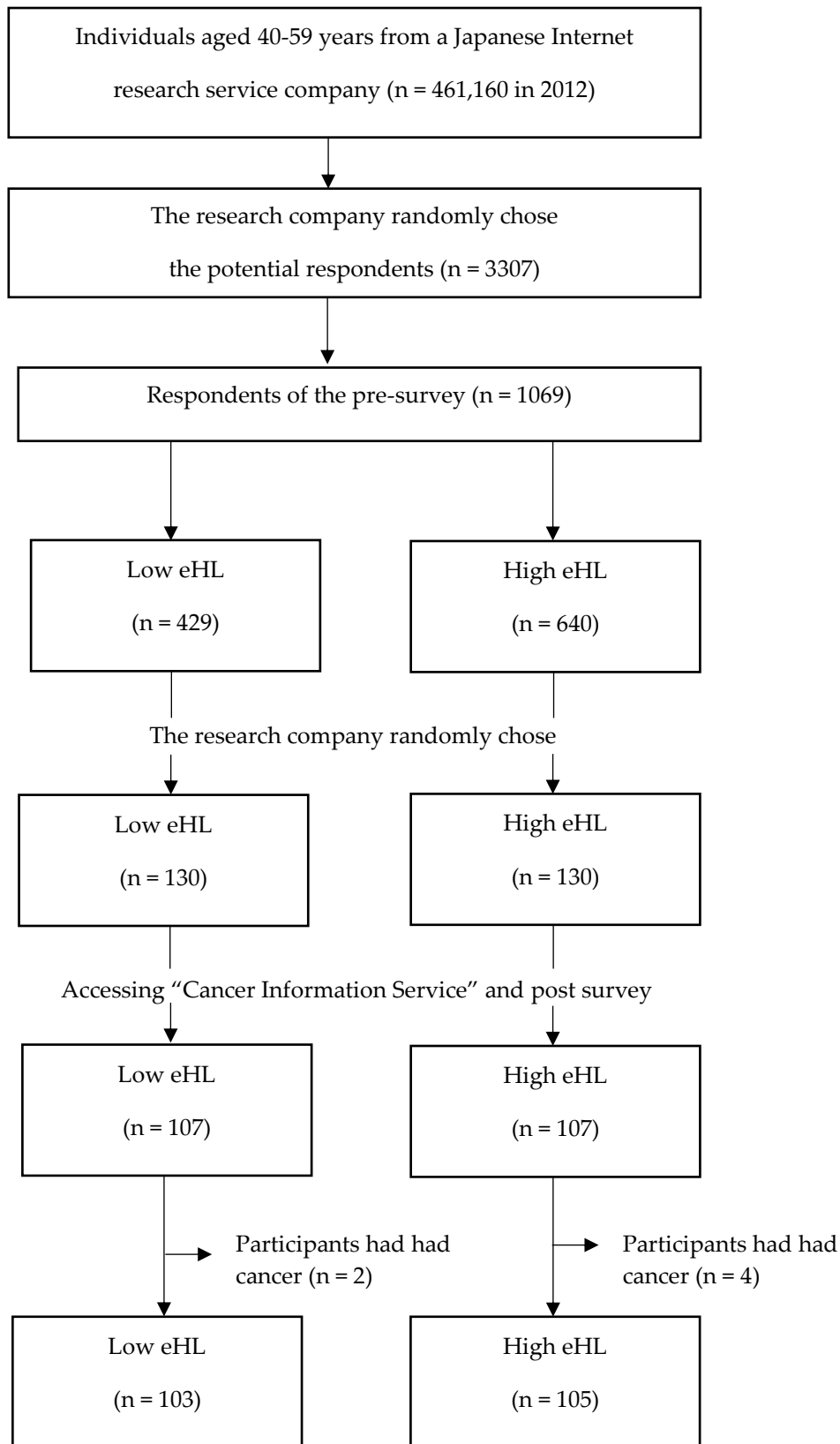

Abbreviations: eHL: eHealth literacy

**Figure S1.** Flowchart of the participants.

Supplement: Supplementary file 1 [file ijerph-17-03302-s001.pdf]
